# Congenital Chloride Diarrhea: Diagnosis by Easy-Accessible Chloride Measurement in Feces

**DOI:** 10.1155/2016/2519498

**Published:** 2016-08-22

**Authors:** C. Gils, M.-C. Eckhardt, P. E. Nielsen, M. Nybo

**Affiliations:** ^1^Department of Clinical Biochemistry and Pharmacology, Odense University Hospital, Odense, Denmark; ^2^Hans Christian Andersen Children's Hospital, Odense University Hospital, Odense, Denmark

## Abstract

*Background.* Congenital chloride diarrhea (CCD) is an autosomal recessive disorder caused by mutations in the genes encoding the intestinal Cl^−^/HCO_3_^−^ exchanger and is clinically characterized by watery, profound diarrhea, electrolyte disturbances, and metabolic alkalosis. The CCD diagnosis is based on the clinical symptoms and measurement of high chloride concentration in feces (>90 mmol/L) and is confirmed by DNA testing. Untreated CCD is lethal, while long-term clinical outcome improves when treated correctly.* Case Presentation*. A 27-year-old woman had an emergency caesarian due to pain and discomfort in gestational week 36 + 4. The newborn boy had abdominal distension and yellow fluid per rectum. Therapy with intravenous glucose and sodium chloride decreased his stool frequency and improved his clinical condition. A suspicion of congenital chloride diarrhea was strongly supported using blood gas analyzer to measure an increased chloride concentration in the feces; the diagnosis was confirmed by DNA testing.* Discussion*. Measurement of chloride in feces using an ordinary blood gas analyzer can serve as a preliminary analysis when congenital chloride diarrhea is suspected. This measurement can be easily performed with a watery feces composition. An easy-accessible chloride measurement available will facilitate the diagnostics and support the initial treatment if CCD is suspected.

## 1. Introduction

Congenital chloride diarrhea (CCD) is an autosomal recessive disorder caused by mutations in the genes encoding the intestinal Cl^−^/HCO_3_^−^ exchanger and is clinically characterized by profound watery diarrhea, electrolyte disturbances, and metabolic alkalosis [1]. CCD has been reported in approximately 250 children worldwide [1]. Geographically, these case reports have been evenly distributed on 6 continents, but in Poland, Finland, and countries around the Persian Gulf the disease is more frequent due to a founder effect [2].

Basically, a defect in the intestinal Cl^−^/HCO_3_^−^ exchanger causes defect intestinal absorption of Cl^−^ and secretion of HCO_3_^−^. The epithelial Cl^−^/HCO_3_^−^ exchanger is coupled with the Na^+^/H^+^ exchanger, which is secondarily affected. These defects cause gastrointestinal loss of both NaCl and fluid and result in a watery chloride diarrhea [1]. If these electrolyte disturbances are not corrected, the renin-angiotensin system will be activated resulting in Na^+^ reabsorption and K^+^ secretion in the distal colon and distal tubuli of the kidney. Overall, laboratory findings of CCD will include hyponatremia, hypochloremia, metabolic alkalosis, and hypokalemia [1]. Clinically, the children can present with large amount of watery diarrhea, lack of meconium, weight loss, distended abdomen, and pain [1, 3]. The treatment of CCD includes life-long oral replacement of the fecal loss of Na^+^, K^+^, Cl^−^, and water [1].

The disease can be revealed during pregnancy by intrauterine onset of watery diarrhea, which is visible on* in utero* sonography as polyhydramnios and dilated bowel loops of the fetus [1, 4]. These characteristics are visible at the end of second trimester, where the distended bowel appears like a honeycomb in the ultrasonic investigation [4].

The diagnosis of CCD is generally based on the clinical presentation and the fecal Cl^−^ concentration (>90 mmol/L) measured after correction of the fluid and electrolyte depletion [5]. Also, genetically testing for CCD by mutation analysis is performed [5].

Untreated, CCD will result in acute and chronic dehydration and renal impairment, a condition that is lethal [6]. Because the long-term clinical outcome improves considerably with adequate treatment, early diagnosis of this disease is crucial. Unfortunately, a rapid diagnosis is impeded by time-consuming analyses, and we therefore here present a typical case of a patient with CCD and an easy-accessible measurement of chloride in feces using an ordinary blood gas analyzer and the challenges associated with the use of nonstandardized laboratory methods.

## 2. Case Presentation

A 27-year-old woman, gravida 4, para 3, was referred to our hospital, a specialized hospital for gastrointestinal surgery in the newborn period, at gestational age 33 + 1 because prenatal ultrasonic investigation had showed small bowel dilatation of the fetus and polyhydramnios indicating fetal intestinal obstruction.

At 36 + 4 weeks of gestation an emergency caesarean section was performed because of progressive polyhydramnios, causing maternal discomfort and pain.

The newborn boy had a birthweight of 2915 g, length of 49 cm, and head circumference of 34 cm. Apgar scores were 10/1 and 10/5. Arterial umbilical cord blood analysis performed immediately after birth showed pH 7.30 (ref. > 7.10) and base excess −0.9 mmol/L (ref. > −10). The boy had abdominal distension and secreted thin yellow fluid per rectum but was doing well. Abdominal X-ray investigation at the age of 4, 16, and 18 hours, a contrast enema, and an upper gastrointestinal contrast study were indicating malrotation of the intestine. A routine presurgical echocardiography showed patent foramen ovale, patent ductus arteriosus, and normal cardiac structures.

Following this, a capillary blood sample showed hemoglobin 9.4 mmol/L (ref. 7.0–11.5), lactate 1.6 mmol/L (ref. 0.5–1.6), normal C-reactive protein, pH 7.36 (ref. 7.35–7.45), base excess −0.7, sodium 140 mmol/L (ref. 137–145), and plasma glucose 5.2 mmol/L. The boy was withheld from enteral nutrition and received intravenous glucose 10% con sodium (20 mmol/L) as infusion. He continuously produced yellow fluid per rectum and did not have passed meconium.

On day 5, a laparotomy was performed revealing malrotation of the intestine. At the same time, blood analyses revealed hyponatremia, which was corrected with intravenous sodium chloride infusion.

Despite surgical correction of the intestinal malrotation, the boy did not gain weight, and he still had abdominal distension, aspirates, and frequent thin stools. He was breastfed and supplied with intravenous fluids. On day 10, the breastfeeding was sufficient and intravenous fluid containing glucose and sodium chloride was stopped.

Over the next days he lost weight and looked pale and chronically sick. Blood analyses showed hyponatremia, potassium in normal range, metabolic alkalosis with pH 7.47, and base excess +5.7. Changing his nutrition was tried without effect. The only clinical effort was achieved by correcting his hyponatremia.

The medical history with prenatal polyhydramnios and bowel distension, lack of meconium, profuse diarrhea, hyponatremia, and metabolic alkalosis led to suspicion of congenital chloride diarrhea. A stool sample was therefore investigated in the hospital laboratory on a blood gas analyzer showing a high fecal chloride concentration of 98 mmol/L.

The diagnosis was subsequently confirmed by DNA analysis displaying a homozygous mutation in the* SLC26A3* gene. This mutation is the well-known Polish founder mutation: c.2024-2026dup, an in-frame duplication.

Both parents were, in this case, healthy and of Polish origin. The three siblings are all healthy. The mother could tell about a family member who had a child who died at the age of 1 week. This child also had clinical symptoms of congenital chloride diarrhea.

## 3. Discussion

Clinically, the CCD diagnosis should be considered in any newborn with large amount of watery diarrhea from birth, dehydration, weight loss, and lack of meconium [5]. Congenital bowel obstruction occurs in intestinal atresia and several other conditions, which also will appear on prenatal ultrasonography as polyhydramnios and dilated bowels [7]. Therefore, these conditions are all prenatally relevant differential diagnoses [4, 7].

However, previous case reports have described prenatal diagnosis of CCD using color Doppler sonography to show the passage of diarrhea [4] and with an ultrasound image of the fetal pelvis showing fluid-filled rectum [7].

The diagnosis is based on the clinical symptoms and measurement of high chloride concentration in the feces, exceeding 90 mmol/L [1, 6]. Although DNA analysis is also available, the feces investigation is the standard procedure in the diagnostics [6]. Untreated, CCD is often lethal, whereas adequate treatment of the disease will improve clinical outcome in terms of normal growth and development [6]. This emphasizes the importance of early diagnosis and treatment of CCD.

Here, we describe a very easy way of measuring the fecal chloride concentration available at most laboratories, namely, using a conventional blood gas analyzer.

The sample was analyzed on the blood gas analyzer ABL800 FLEX (Radiometer, Denmark), which is validated and used for measurement of electrolytes (potassium, sodium, and chloride) by a potentiometric principle using an ion-selective electrode whose sensing element is a membrane containing an ion specific carrier. The instrument is used for routine measurements in plasma, serum, whole blood, capillary blood, spinal fluid, and other special body fluids, and a matrix effect therefore cannot be excluded in this case. To minimize a potential matrix effect, the sample was centrifuged several times. A coagulation filter placed between the sample and the instrument was used to protect the instrument. The sample was analyzed twice, but the result was not confirmed by other standard chemistry analysis principles.

In general, diarrheal chloride could at any given suspicion be used as an easy-accessible rule-out biomarker for this disease. The method presented here is very simple, accessible in most laboratories, and reliable. The time gained to implement the correct treatment using the simple and highly reliable fecal Cl measurement described here is evident from the fact that the chloride measurement is available in a matter of hours, while the targeted or whole exome sequencing takes several weeks.

Other inherited secretory diarrhea diseases are relevant differential diagnoses in patients with CCD and should be considered initially [1]. In such cases, alkaline feces will exclude the possibility of CCD [1, 8]. At our laboratory, pH in feces is measured by use of indicator paper with a reference interval of pH 7-8. A result of pH > 8 will reflect alkaline feces. Again, pH measurement using a blood gas analyzer would be an easy and reproducible way to achieve this result. In this case, a pH measurement was not performed but in future cases it will be important to do so.

The diagnosis of CCD might be difficult since it is a rare disease, and it will strongly depend on the clinician, who must be aware of the clinical symptoms. Hence, a number of patients are believed to be undiagnosed [2, 9]. Again, an easy-accessible and cheap measurement principle is important in order to facilitate improved diagnostics in these children.

Although CCD might be suspected based on the clinical presentation, there can be situations where the diarrheal chloride concentration does not exceed the cut-off value of 90 mmol/L because of feces dilution by urine or because of reduction in feces amount due to volume and salt depletion [1, 6]. In such cases, repeated testing for chloride in diarrhea after correction of the fluid and electrolyte deficiency is necessary [5].

In conclusion, we here report on a case, where CCD was initially diagnosed based on increased chloride concentration in diarrhea. Because early diagnosis of this disease leads to significant improvement of survival and long-term clinical outcome, it is mandatory that the clinician is aware of the pathogenesis and also has an easy-accessible chloride measurement available. If the measurement of diarrheal chloride, as in this case, is not validated, the result must be interpreted with caution. However, the method is simple, easy-accessible, and highly reliable and can be used as a guide until the result can be confirmed by genetic testing. In this case, the result, although correctness can be questionable, supports the clinical suspicion of congenital chloride diarrhea and thereby supports the effect of correcting the patient's hyponatremia.
